# Supplementary material for: Silanized Graphene Oxide-Supported Pd Nanoparticles and Silicone Rubber for Enhanced Hydrogen Elimination
Source: Materials (Basel). 2022 Jun 29;15(13):4578. doi: 10.3390/ma15134578 (PMC9267170; doi:10.3390/ma15134578)
Supplement: Supplementary file 1 [file materials-15-04578-s001.zip › materials-1768834-supplementary.pdf]

materials

MDPI

Article

Silanized Graphene Oxide Supported Pd Nanoparticles and Silicone Rubber for Enhanced Hydrogen Elimination

Yu Wang [1], Tao Xing [2,*], Yongqi Deng [1], Kefu Zhang [1], Yihan Wu [1] and Lifeng Yan [1,*]

1 Department of Chemical Physics, University of Science and Technology of China, Hefei 230026, China; wy1998@mail.ustc.edu.cn (Y.W.); ddyyqq@mail.ustc.edu.cn (Y.D.); zkefu@mail.ustc.edu.cn (K.Z.); yihanwu@mail.ustc.edu.cn (Y.W.)
2 Institute of System and Engineering, China Academy of Engineering Physics, 64 Mianshan Road, Mianyang 621900, China
* Correspondence: 412xingt@caep.cn (T.X.); lfyan@ustc.edu.cn (L.Y.); Tel.: +86-551-63606853 (L.Y.)

Citation: Wang, Y.; Xing, T.; Deng, Y.; Zhang, K.; Wu, Y.; Yan, L. Silanized Graphene Oxide Supported Pd Nanoparticles and Silicone Rubber for Enhanced Hydrogen Elimination. *Materials* **2022**, *15*, 4578. https://doi.org/10.3390/ma15134578

Academic Editor: Abbes Tahraoui

Received: 28 May 2022
Accepted: 27 June 2022
Published: 29 June 2022

Publisher's Note: MDPI stays neutral with regard to jurisdictional claims in published maps and institutional affiliations.

Copyright: © 2022 by the authors. Licensee MDPI, Basel, Switzerland. This article is an open access article distributed under the terms and conditions of the Creative Commons Attribution (CC BY) license (https://creativecommons.org/licenses/by/4.0/).

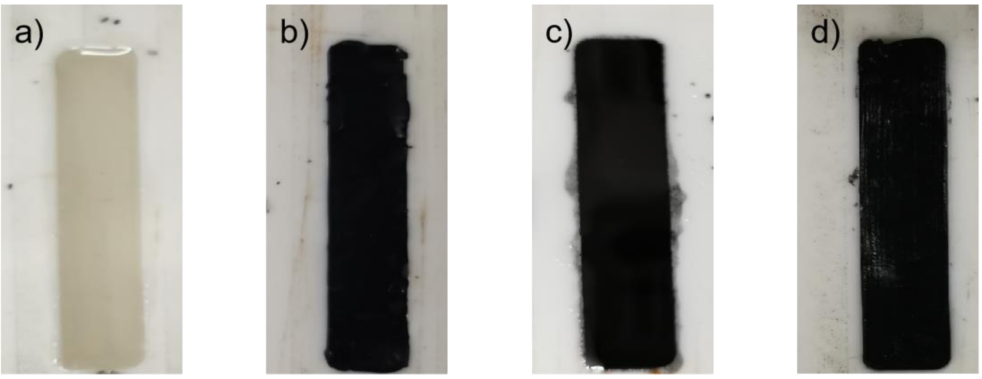

Figure S1. The physical images of (a) pure SR, (b) G1, (c) G2 and (d) G3.

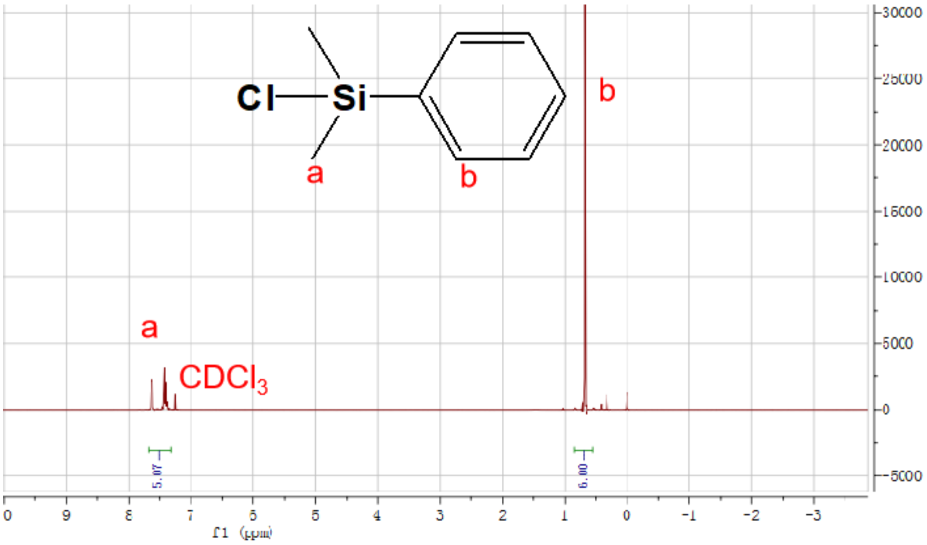

Figure S2. ^{1}H NMR spectrum of chlorodimethyl(phenyl)silane in CDCl3.

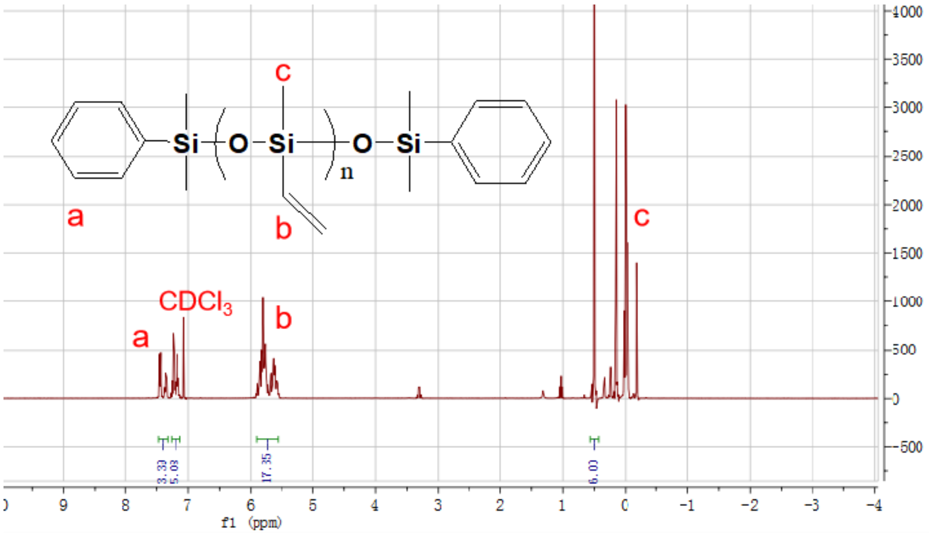

Figure S3. ^{1}H NMR spectrum of the product in CDCl3.

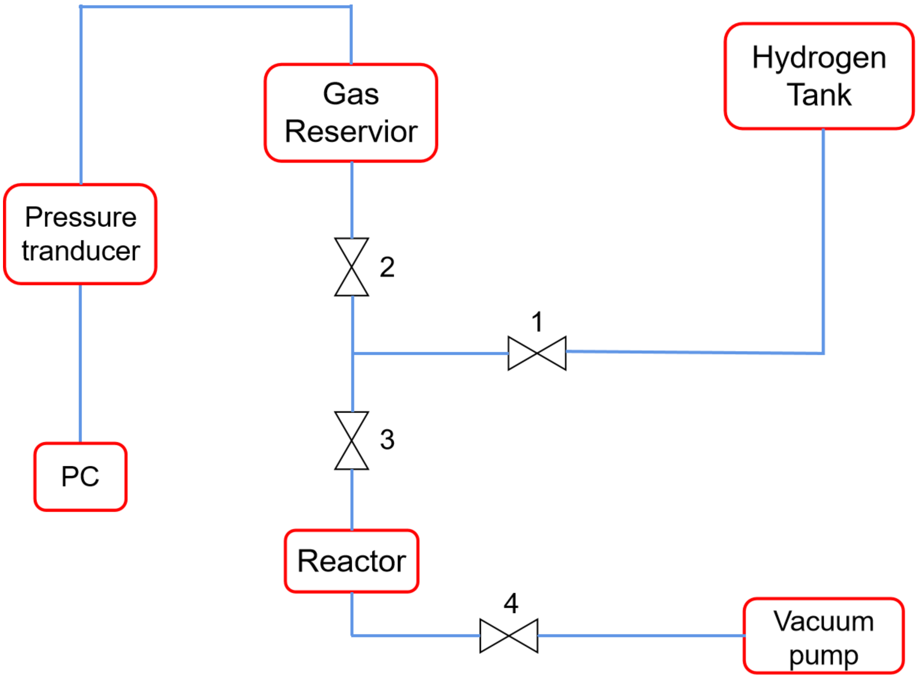

Figure S4. Schematic illustration of pure hydrogen test apparatus.

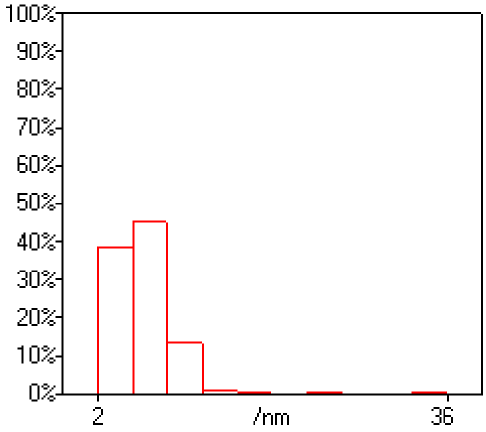

Figure S5. The size of 200 Pd nanoparticles randomly counted.
